# Fructooligosaccharides and *Aspergillus* enzymes increase brain GABA and homocarnosine by modulating microbiota in adolescent mice

**DOI:** 10.1038/s41538-025-00383-1

**Published:** 2025-04-03

**Authors:** Jason D. Braga, Yongshou Yang, Tomoka Nagao, Norihisa Kato, Noriyuki Yanaka, Kyoichi Nishio, Masamichi Okada, Manabu Kuroda, Shotaro Yamaguchi, Thanutchaporn Kumrungsee

**Affiliations:** 1 Program of Food and AgriLife Science, Graduate School of Integrated Sciences for Life, Hiroshima University, Hiroshima, Japan; 2 Institute of Food Science and Technology, College of Agriculture, Food, Environment and Natural Resources, Cavite State University, Cavite, Philippines; 3 School of Life Sciences, Anhui University, Hefei, China; 4 Amano Enzyme Inc., Nagoya, Japan; 5 Smart Agriculture, Graduate School of Innovation and Practice for Smart Society, Hiroshima University, Hiroshima, Japan

**Keywords:** Microbiome, Small molecules

## Abstract

Recent research suggests that dietary prebiotics, probiotics, or healthy fecal-microbiota transplantation attenuate gut microbiota dysbiosis and ameliorate neurological disorders, in which gut-microbiota-derived γ-aminobutyric acid (GABA) has gained much attention as one of key mediators in the gut-brain axis. Although it is widely accepted that prebiotics and probiotics induce gut and brain GABA production via modulating gut microbiota, only evidence of probiotics has been solidly demonstrated while this evidence of prebiotics is scarce. Here, we demonstrated that prebiotic fructo-oligosaccharides and *Aspergillus*-derived enzymes elevated gut and brain GABA concentrations by modulating gut microbiota. Interestingly, we found that the prebiotic and enzymes increased a brain-specific dipeptide, homocarnosine. Gut GABA levels were found correlated with brain GABA/homocarnosine levels. *Parabateroides*, *Akkermansia, Muribaculum, Hungatella*, *Marvinbryantia, Flavonifractor*, and *Incertae_sedis* exhibited a positive correlation with gut GABA and brain GABA/homocarnosine levels, while *Blautia*, *Unclassified_Lachnospiraceae*, *Colidextribacter*, Acetatifactor, *Roseburia*, *Unclassified_Oscillospiraceae*, *Romboutsia*, and *Eubacterium_coprostanoligenes* exhibited a negative correlation with those levels.

## Introduction

γ-Aminobutyric acid (GABA) is a principle inhibitory neurotransmitter that mediates neuronal inhibition in the brain^1^. Imbalance between excitation and inhibition, such as excessive neuronal excitation or insufficient neuronal inhibition, can result in various neuronal disorders. For instance, low brain GABA levels are often found in epileptic patients^2,3^. Recent research suggests that low brain GABA concentrations are not only associated with epilepsy but also with other brain diseases, such as Alzheimer’s disease, autism spectrum disorder, and depression^4–7^. Therefore, rectifying insufficient GABA-neuronal inhibition via increasing brain GABA concentrations or enhancing GABA-inhibitory signals in the brain is an approach to prevent or treat those neuronal disorders.

Besides anticonvulsant drugs, such as vigabatrin, that are used to increase brain GABA levels and ameliorate epileptic seizures, circulating or peripheral GABA is a potent effector enhancing GABA-inhibitory signals in the brain^8^. Recent research proposes that GABA is a possible mediator in the gut-brain axis mediating peripheral signals to the brain^9^. In addition to an exogenous source, such as GABA-containing foods and dietary GABA, an endogenous source of peripheral GABA is hypothesized to be mainly attributed to gut microbiota. This hypothesis is supported by evidence showing that germ-free and antibiotic-treated mice exhibited a strong decrease in blood and cecal GABA levels and GABA is the most changed blood metabolites after fecal microbiota transplantation in humans^10–13^.

Accumulating evidence demonstrates that ingestion of GABA-producing probiotics, such as *Lactobacillus* and *Bifidobacterium*, significantly increased peripheral (blood and gut) GABA concentrations^14–17^, brain GABA concentrations^18–23^, or brain GABA receptor expressions^22,24,25^, with amelioration of neurological dysfunctions, such as depression and anxiety-like behaviors^14,19^, epileptic seizures^16,20,24^, and abdominal pain^15^. Since prebiotics potentially modulate gut microbiota composition and increase an abundance of beneficial probiotics, the idea that prebiotics induce production of gut microbiota-derived metabolites, including the neurotransmitter GABA, and to be the mediators communicating with the brain is widely accepted. However, to the best of our knowledge, there are only few studies demonstrating an increase in peripheral and brain GABA concentrations by dietary prebiotics^26–29^. Moreover, there is no evidence showing a direct association between prebiotics, gut microbiota, gut GABA, and brain GABA.

Fructo-oligosaccharides (FOS), non-digestible oligosaccharides, are the typical and well-known prebiotics extensively studied for their effects and shown to benefit human health^30^. In 1995, the prebiotic concept was first defined as “a non-digestible food ingredient that beneficially affects the host by selectively stimulating the growth and/or activity of one or a limited number of bacteria already resident in the colon”^30^. In recent years, this concept has been broadened and redefined as “a non-digestible compound that, through its metabolization by microorganisms in the gut, modulates the composition and/or activity of the gut microbiota, thus, conferring a beneficial physiological effect on the host”^30^. This expanded prebiotic concept now includes compounds beyond non-digestible oligosaccharides. Recently, a range of substrates that impact microorganisms, induce microbial metabolite production, and provide health benefits have also been considered as prebiotics; these emerging prebiotics include some fatty acids, human milk oligosaccharides, and plant polyphenols^30^. To date, evidence for these emerging prebiotics is scarce relative to typical oligosaccharide prebiotics, and more studies evaluating their health benefits are required to confirm their prebiotic status.

Our recent studies have shown that the intake of digestive enzymes derived from *Aspergillus*—including proteases, cellulases, and lipases—increased the abundance of probiotics, such as *Bifidobacterium* and *Lactobacillus*, altered the gut microbiota composition, and stimulated the production of microbial metabolites, including cecal short-chain fatty acids (SCFAs) and amino acids like taurine, ornithine, phenylalanine, cysteine, and GABA^27,31–33^. These SCFAs and amino acids were either present in trace levels or undetectable in mice not receiving *Aspergillus*-derived enzymes, suggesting that these metabolites were possibly produced or released by gut microbiota. Given our findings that their enzymatic activities are crucial for exerting those prebiotic-like effects and likely resist rodent host digestion^27^, these enzymes might indirectly promote the growth of probiotics or alter composition of gut microbiota by possibly digesting undigested macronutrients in the large intestine, thereby increasing the availability of energy substrates for microbial growth. These observed effects and properties suggest that *Aspergillus*-derived enzymes may serve as useful substrates with prebiotic-like effects. While the concept of using enzymes as supplements to modulate gut microbiota and promote health is new in human contexts, exogenous enzymes, commonly derived from such as fungi like *Aspergillus*, are widely used in the feed industry as an alternative to antibiotics to improve animal performance^34–36^. These enzymes are used to degrade feed components that are resistant to endogenous enzymes, enhancing the hydrolysis of undigested macronutrients and releasing nutrients (e.g., glucose, amino acids, and fatty acids) that in turn stimulate probiotic growth, alter gut microbiota profiles, and induce microbial metabolite production^34–36^.

In the present study, we aimed to investigate effects of the typical FOS prebiotic and *Aspergillus*-derived enzymes, lipase (AL) and protease (AP), on inducing gut GABA production and brain GABA elevation by modulating gut microbiota in adolescent mice. Since GABA metabolism links to its GABA-containing peptide, homocarnosine (GABA-L-histidine), metabolism^9^, changes in GABA levels may alter homocarnosine levels. Therefore, in the present study, we also determined effects of FOS and the enzymes on homocarnosine levels in the brain.

## Results

### Food intake, body weight, and cecum and tissue weights

To understand physical effects of the prebiotic FOS and enzyme supplementations on mice, food intake, body weight, and tissue weights were measured after 4 weeks of feeding. As compared with the control (Ctrl) group, all the supplement groups (FOS, AL, and AP) had no effects on final body weight, food intake, fat tissue weight, and tibialis anterior skeletal muscle weight (Table 1). Among the supplement groups, FOS significantly increased the cecum content wet weight to 3.3-fold (0.14 ± 0.02 (Ctrl) vs. 0.46 ± 0.05 (FOS) g, *p* < 0.05), while AL and AP slightly increased the cecum content wet weight, but no significant differences (Table 1).

**Table 1:** Effects of prebiotics on body weight, food intake, cecal contents, and tissue weights

	Ctrl	FOS	AL	AP
Initial weight (g)	30.7 ± 0.5	31.8 ± 0.4	31.1 ± 0.7	31.2 ± 0.5
Weight gain in 1 month (g)	6.9 ± 0.3	8.2 ± 0.3	7.4 ± 0.4	7.5 ± 0.4
Final weight (g)	37.6 ± 0.9	40.0 ± 0.7	38.5 ± 1.0	38.7 ± 1.4
Food intake in 1 month (g)	179.5 ± 1.0	184.3 ± 0.9	170.5 ± 0.9	179.9 ± 1.0
Cecal content (g)	0.14 ± 0.02^a^	0.46 ± 0.05^b^	0.21 ± 0.03^a^	0.16 ± 0.01^a^
White fat (g)	0.61 ± 0.06	0.59 ± 0.06	0.78 ± 0.14	0.70 ± 0.12
Tibialis anterior (TA, g)	0.06 ± 0.002	0.06 ± 0.002	0.06 ± 0.002	0.07 ± 0.003

Values are expressed as the means ± SE (*n* = 8). Statistical significance between control group and each treatment group was determined by One-Way ANOVA following the Dunnett’s multiple comparison test at 95% level of confidence (*p* < 0.05). Superscripts with different letters were significantly different at *p* < 0.05. No superscript means no significant difference between the control group and each treatment group.

*Ctrl* control, *FOS* fructo-oligosaccharides, *AL* Aspergillus-derived lipase, and *AP* Aspergillus-derived protease.

### Effects of the prebiotic FOS and enzyme supplementations on GABA and homocarnosine levels in cecum, plasma, and brain

**Fig. 1:**
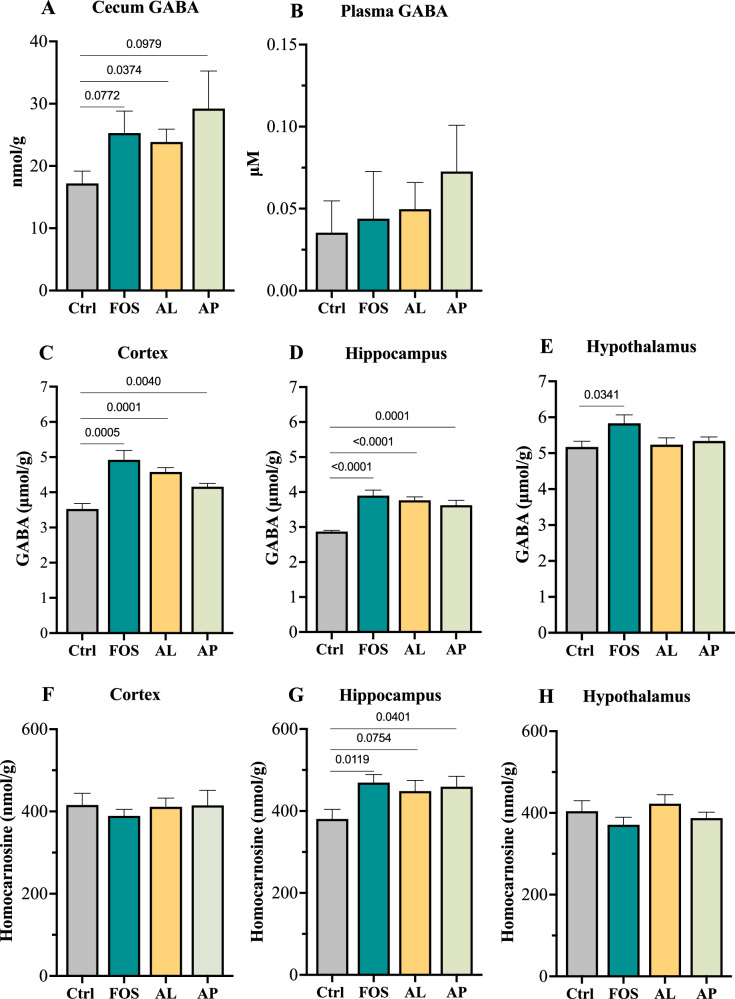
Effect of prebiotics on GABA and homocarnosine levels in cecal content, plasma, and brain. **A** cecal GABA; **B** plasma GABA; **C**–**E** brain GABA levels; **F**–**H** brain homocarnosine levels (*n* = 8). Numbers above the bars indicate *p* values analyzed using a two-tailed unpaired *t*-test with *p* < 0.05 indicating statistical significance.

To determine effects of the prebiotic FOS and enzyme supplementations on GABA and homocarnosine levels in gut and brain, cecum, plasma, and brain samples were analyzed. As compared with the Ctrl group, both a typical prebiotic (FOS, 1.5-fold, *p* = 0.0772) and the enzymes (AL (1.4-fold, *p* = 0.0374) and AP (1.7-fold, *p* = 0.0979)) increased GABA concentrations in the cecal content (Fig. 1A). In blood, an elevation of GABA was not observed across all supplement groups (Fig. 1B). In the brain, all FOS and enzyme supplementations significantly increased GABA concentrations in cortex and hippocampus, while only FOS exhibited a significant increase in GABA levels in hypothalamus (Fig. 1C–E). Since GABA is a constituent amino acid of the predominant brain peptide, homocarnosine (GABA-L-histidine), we measured brain levels of this peptide. Interestingly, we found that all FOS and enzyme supplementations markedly increased homocarnosine levels in hippocampus (380.4 ± 23.7 (Ctrl) vs. 469.3 ± 19.6 (FOS) nmol/g, *p* = 0.0119; vs. 448.4 ± 26.3 (AL) nmol/g, *p* = 0.0754; or vs. 459.2 ± 25.5 (AP) nmol/g, *p* = 0.0401, Fig. 1F–H), while no effects on those levels in cortex and hypothalamus (Fig. 1F–H). Taken together, these results indicate that dietary intake of the prebiotic FOS and the *Aspergillus*-derived enzymes potentially induced the elevation of gut GABA, brain GABA, and brain homocarnosine.

### Changes in cecal microbiota by the prebiotic FOS and enzyme supplementations

**Fig. 2:**
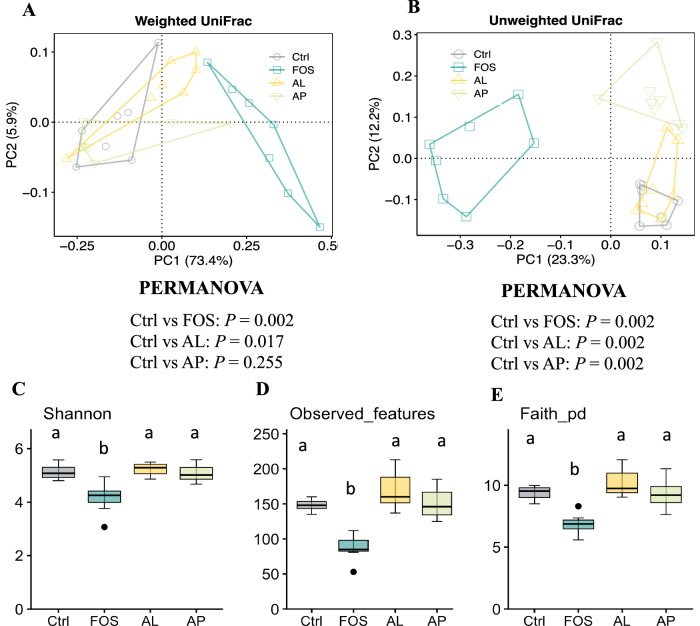
Effect of prebiotics on cecal microbial composition and alpha diversity. PCoA of weighted (**A**) and unweighted UniFrac (**B**) and PERMANOVA analysis to compare the gut microbiome profiles of the experimental groups. The alpha diversity of the gut microbiota within samples was measured by Shannon index (**C**) observed features (**D**) and Faith PD (**E**). Data are presented as a boxplot with median and min–max whiskers (*n* = 7–8). The dots (•) in the boxplots are outliers. Different letters above the bars indicate significant differences when compared to the Ctrl group (Dunnett’s test). *p* < 0.05 was considered statistically significant. Ctrl Control, FOS fructo-oligosaccharides, AL *Aspergillus*-derived lipase, and AP *Aspergillus*-derived protease.

In order to determine the involvement of gut microbiota in the production of gut GABA, 16S rRNA analysis by gene sequencing was carried out. Unweighted and weighted UniFrac PCoA and PERMANOVA analyses exhibited significant different microbial compositions between the Ctrl and FOS groups and the Ctrl and AL groups in weighted UniFrac (Fig. 2A, *p* < 0.05) and between all supplement groups against the Ctrl group in unweighted UniFrac (Fig. 2B, *p* < 0.05). The alpha-diversity indices indicated less richness in bacterial diversity in the FOS group (Fig. 2C–E, *p* < 0.05).

**Fig. 3:**
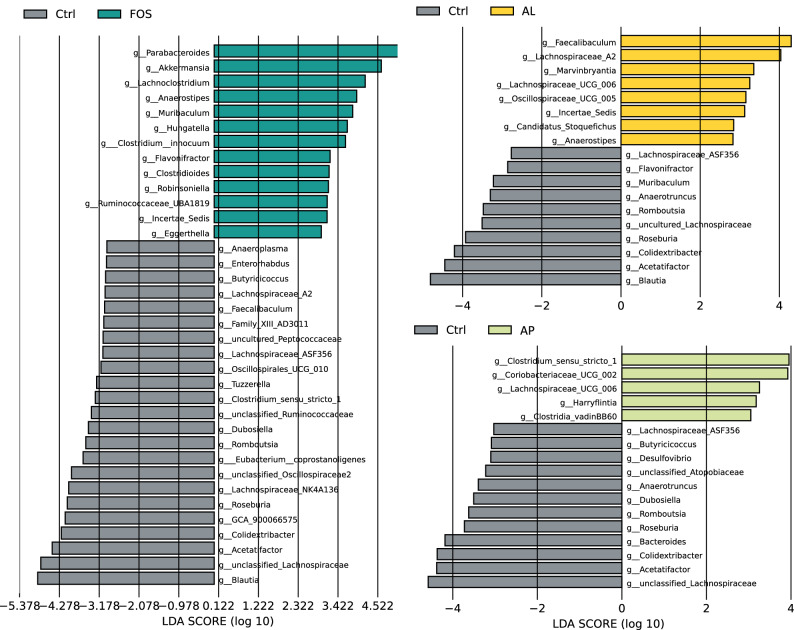
LEfSe analysis on the comparison of different bacterial taxa between the control and supplement groups. The two-tailed nonparametric Kruskal–Wallis test was used to evaluate the significant differences in taxa between the Ctrl group and each prebiotic group (*p* < 0.05, *n* = 7–8). Ctrl Control, FOS fructo-oligosaccharides, AL *Aspergillus*-derived lipase, and AP *Aspergillus*-derived protease.

**Fig. 4:**
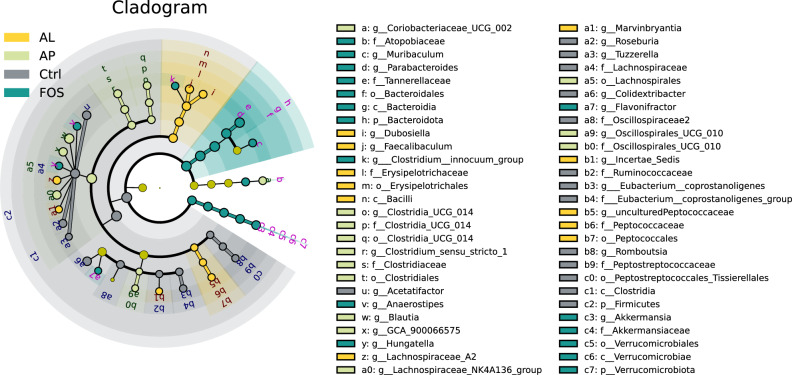
Cladogram analysis. The gray, green, yellow, and light green nodes in the phylogenetic tree represent microbial taxa that play an important role in the Ctrl, FOS, AL, and AP groups, respectively. Ctrl Control, FOS fructo-oligosaccharides, AL *Aspergillus*-derived lipase, and AP *Aspergillus*-derived protease.

Linear discriminant analysis (LDA) effect size (LEfSe) showed that the top five enriched genera in the FOS, AL, and AP groups are *Parabacteroides*, *Akkermansia*, *Lachnoclostridium*, *Anaerostipes*, and *Muribaculum*; *Faecalibaculum*, *Lachnospiraceae_A2*, *Marvinbryantia*, *Lachnospiraceae_UCG_006*, and *Oscillospiraceae_UCG_005*; and *Clostridium_sensu_stricto_1*, *Coriobacteriaceae_UCG_002*, *Lachnospiraceae_UCG_006*, *Harryflintia*, and *Clostridia_vadinBB60*, respectively (Fig. 3). The top five reduced genera in the FOS, AL, and AP groups are *Blautia*, *Unclassified_Lachnospiraceae*, *Acetatifactor*, *Colidextribacter*, and *GCA_900066575*; *Blautia*, *Acetatifactor*, *Colidextribacter*, *Roseburia*, and *Uncultured_Lachnospiraceae*; and *Unclassified_Lachnospiraceae*, *Acetatifactor*, *Colidextribacter*, *Bacteroides*, and *Roseburia*, respectively (Fig. 3). Cladograms illustrating changes in microbial taxa at all hierarchical levels between the control and supplement groups were shown in Fig. 4.

**Fig. 5:**
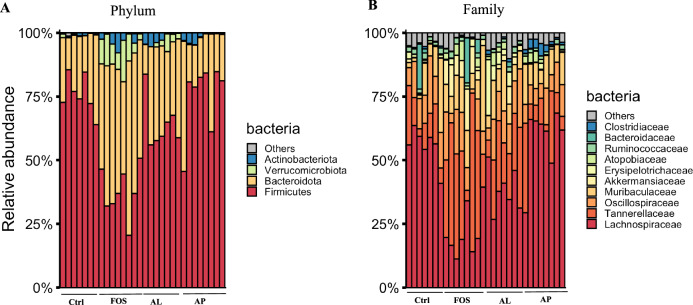
Effect of prebiotics on cecal microbial composition at phylum (**A**) and family (**B**) levels. Ctrl Control, FOS fructo-oligosaccharides, AL *Aspergillus*-derived lipase, and AP *Aspergillus*-derived protease.

At the phylum level (Fig. 5A), FOS significantly decreased the relative abundance of *Firmicutes*, while the relative abundance of *Bacteriodota* and *Verrucomicrobiota* were enriched, as compared to Ctrl (Fig. 5A, *p* < 0.05). These changes in the phyla of the FOS group affected at the family level where the relative abundance of *Lachnospiraceae* (phylum: *Firmicutes*) was significantly decreased and *Tannerellaceae* (phylum: *Bacteriodota*) was significantly increased (Fig. 5B, *p* < 0.05). For AL and AP groups, bacterial phyla were not affected (Fig. 5A), but bacterial families were significantly affected by these *Aspergillus*-derived enzymes (Fig. 5B). In the AL group, the relative abundance of *Lachnospiraceae* (phylum: *Firmicutes*) was decreased and the relative abundance of *Erysipelotrichaceae* (phylum: *Firmicutes*) was increased (Fig. 5B, *p* < 0.05). In the AP group, the abundance of *Clostridiaceae* (phylum: *Firmicutes*) was enriched (Fig. 5B, *p* < 0.05).

**Fig. 6:**
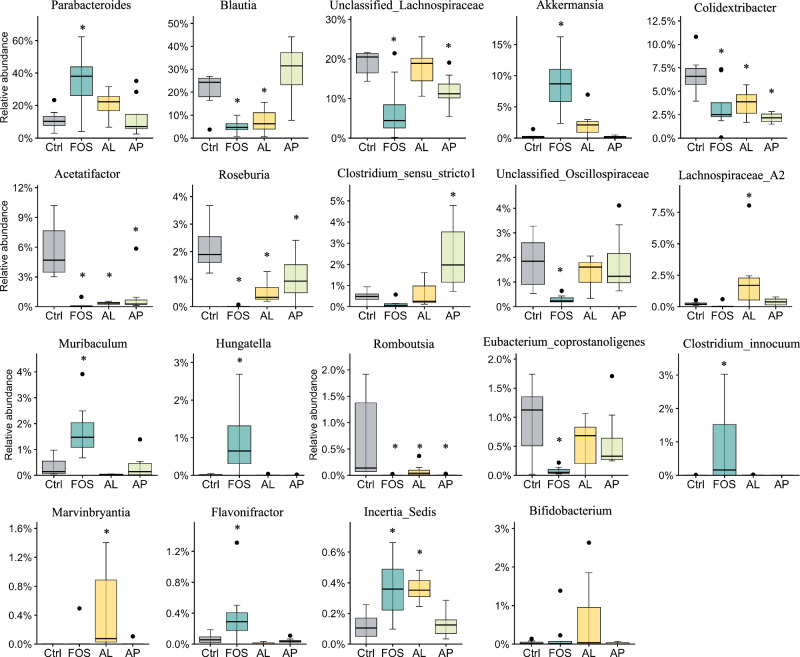
Effect of prebiotics on cecal microbial composition at genus levels. Data are presented as a boxplot with median and min–max whiskers (*n* = 7–8). The dots (•) in the boxplots are outliers. * *p* < 0.05 means significantly different when compared to the Ctrl group (Dunnett’s test). Ctrl Control, FOS fructo-oligosaccharides, AL *Aspergillus*-derived lipase, and AP *Aspergillus*-derived protease.

At the genus level (Fig. 6), a total of 18 genera showed significant changes (*p* < 0.05) due to the prebiotic FOS and enzyme treatments (Fig. 6). As compared to Ctrl, FOS increased the relative abundance of *Parabacteroides, Akkermansia, Muribaculum, Hungatella, Clostridium_innocuum*, Flavonifractor, and *Incertae_sedis* and decreased the relative abundance of *Blautia*, *unclassified_Lachnospiraceae*, *Colidextribacter*, *Acetatifactor*, *Roseburia*, *Unclassified_Oscillospiraceae*, *Romboutsia*, and *Eubacterium_coprostanoligenes*. In the AL group, a relative abundance of *Lachnospiraceae_A2*, *Marvinbryantia*, and *Incertae_sedis* was enriched, while a relative abundance of *Blautia*, *Colidextribacter*, *Acetatifactor*, *Roseburia*, and *Romboutsia* was decreased. In the AP group, an enriched abundant genus was *Clostridium_sensu_stricto_1* and decreased abundant genera were *Unclassified_ Lachnospiraceae*, *Colidextribacter*, *Acetatifactor*, *Roseburia*, and *Romboutsia*. The relative abundance of the well-known probiotic *Bifidobacterium* was slightly increased in the AL group, but showed no significant difference. The results indicate that FOS and each enzyme induced changes in the different compositions of gut microbiota.

### Correlation of gut and brain GABA and homocarnosine with gut microbiota

**Fig. 7:**
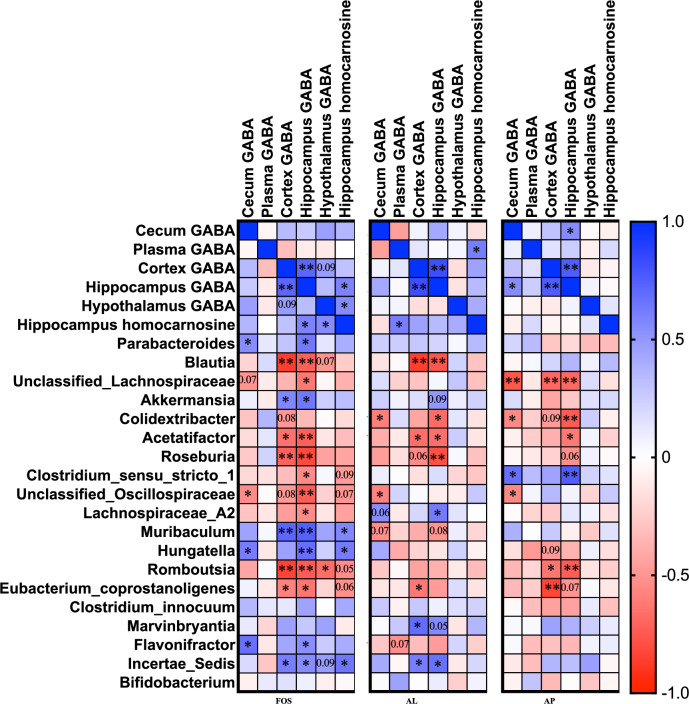
Heatmap of the correlations among the relative abundances of gut microbiota at the generic level, peripheral GABA levels, brain GABA levels, and brain homocarnosine levels under each prebiotic treatment. Spearman correlation analysis was used for the analysis. * *p* < 0.05 means significantly different between the two parameters. Numbers indicate *p* values, which show a tendency correlation (0.05 < *p* < 0.1) between the two parameters. FOS fructo-oligosaccharides, AL *Aspergillus*-derived lipase, and AP *Aspergillus*-derived protease.

To determine association between gut GABA, brain GABA, brain homocarnosine, and gut microbiota under prebiotic FOS or each enzyme treatment, a correlation analysis was conducted with the result shown in Fig. 7. Under the FOS treatment, there is a significant positive correlation between hippocampal GABA levels and hippocampal homocarnosine. Cecal GABA levels showed a significant positive correlation with *Parabateroides*, *Hungatella*, and *Flavonifractor*, but a significant negative correlation with *Unclassified_Oscillospiraceae*. Brain GABA and homocarnosine levels were significantly positively correlated with bacterial genera of *Parabateroides*, *Akkermansia, Muribaculum, Hungatella*, *Flavonifractor*, and *Incertae_sedis*, while significantly negatively correlated with *Blautia*, *Unclassified_Lachnospiraceae*, *Acetatifactor*, *Roseburia*, *Clostridium_sensu_stricto_1*, *Unclassified_Oscillospiraceae*, *Lachnospiraceae_A2*, *Romboutsia*, and *Eubacterium_coprostanoligenes*. Under the AL treatment, plasma GABA levels exhibited a significant positive correlation with hippocampal homocarnosine levels. Cecal GABA levels had a tendency positive correlation with *Lachnospiraceae_A2* (*p* = 0.061), but a significant negative correlation with *Colidextribacter* and *Unclassified_Oscillospiraceae*. Brain GABA levels exhibited a significant positive correlation with *Lachnospiraceae_A2*, *Marvinbryantia*, and *Incertae_sedis*, but a significant negative correlation with *Blautia*, *Colidextribacter*, *Acetatifactor*, *Roseburia*, and *Eubacterium_coprostanoligenes*. Under the AP treatment, there is a significant positive correlation between cecal GABA levels and hippocampal GABA levels. Cecal GABA levels exhibited a significant positive correlation with *Clostridium_sensu_stricto_1*, but a significant negative correlation with *Unclassified_Lachnospiraceae*, *Colidextribacter*, and *Unclassified_Oscillospiraceae*. Brain GABA levels exhibited a significant positive correlation with *Clostridium_sensu_stricto_1*, but a significant negative correlation with *Unclassified_Lachnospiraceae*, *Colidextribacter*, *Acetatifactor*, *Romboutsia*, and *Eubacterium_coprostanoligenes*.

In summary, a positive correlation between peripheral GABA levels and brain GABA or homocarnosine levels was observed under those FOS and enzyme supplementations. In the brain, although not all supplement groups, a positive correlation between GABA levels and homocarnosine levels was observed. Bacterial genera that exhibited a positive correlation with peripheral and brain GABA and homocarnosine levels were *Parabateroides*, *Akkermansia, Muribaculum, Hungatella*, *Marvinbryantia, Flavonifractor*, and *Incertae_sedis*. On the other hand, bacterial genera that exhibited a negative correlation with peripheral and brain GABA and homocarnosine levels were *Blautia*, *Unclassified_Lachnospiraceae*, *Colidextribacter*, Acetatifactor, *Roseburia*, *Unclassified_Oscillospiraceae*, *Romboutsia*, and *Eubacterium_coprostanoligenes*. *Clostridium_sensu_stricto_1* and *Lachnospiraceae_A2* genera exhibited both positive and negative correlations with peripheral and brain GABA and homocarnosine levels.

## Discussion

Emerging research suggests the association among neurological disorders, dyshomeostasis of gut and brain GABA, and dysbiosis of gut microbiota^9,37^. The imbalance of GABA-producing gut bacteria is associated to certain illnesses. For instance, altered abundance of the GABA-producing *Lactobacillus* and *Bifidobacterium* is linked to stress-induced anxiety and depression-related behaviors^25,38^, seizure episodes in patients with epilepsy^39^, and children with attention deficit hyperactivity disorder (ADHD)^40^. In addition, markedly decreases in fecal GABA concentrations and expression of GABA-producing enzyme glutamate decarboxylase in gut microbiota were found in patients with essential tremor^17^. Therefore, it has been proposed that intake of prebiotics and probiotics is a potential approach to treat those neurological disorders. Prebiotics and probiotics are proposed to ameliorate symptoms of neurological disorders by modulating gut microbiota composition that, in turn, producing metabolites, including GABA, that act as mediators in restoring balance of GABA metabolism in the brain via increasing brain GABA levels itself or upregulating GABA receptors.

To date, there is some solid evidence indicating that dietary probiotics induced elevation of peripheral (gut and blood) GABA levels and/or brain GABA levels. However, there is only few studies demonstrating this ability of prebiotics in increasing gut and/or brain GABA levels. Moreover, there is no evidence showing the direct relationship among prebiotics, gut microbiota, gut GABA, and brain GABA. Here, we demonstrated that dietary intake of prebiotic FOS or substrates with prebiotic-like effects, such as the digestive enzymes derived from *Aspergillus*, induced gut GABA production and brain GABA elevation by modulating gut microbiota. In addition, we newly demonstrated that those FOS and enzyme supplementations could induce an elevation of the brain GABA reservoir peptide, homocarnosine. Our findings reveal the direct association among gut GABA, brain GABA, and gut microbiota under the prebiotic FOS and enzyme treatments.

To date, some studies demonstrated an ability of probiotics in increasing peripheral and brain GABA levels. Dietary *Lactobacillus* and *Bifidobacterium* probiotics were found to significantly increase small intestinal and cecal content GABA levels in mice^14,15^. A mixture of *Lactobacillus, Bifidobacterium*, and *Streptococcus* probiotics induced a sharp increase in serum GABA levels in patients with epilepsy^16^. A combination of a *Lactobacillus* probiotic with a galactooligosaccharides prebiotic exhibited a synergistic effect on increasing serum GABA concentrations with the stronger effect under a ketogenic diet^24^. In piglets, *Clostridium* and *Bacillus* probiotics induced an increase in both serum and brain GABA levels^12^. In an in vitro human intestinal microbial ecosystem mimicking model, *Lactobacillus* and *Bifidobacterium* probiotic treatment strongly enhanced GABA production in the colonic media^41^. Supplementing a strong-GABA-producing *Lactobacillus* probiotic significantly increased and restored gut GABA levels in GABA-deficient essential tremor mice^17^, while supplementing a strong-GABA-producing *Lactiplantibacillus* probiotic significantly increased and restored hippocampal brain GABA levels in anxiety-induced mice with anxiety improvement^19^. In addition to increasing or restoring brain GABA level itself^18,20–23^, dietary probiotics, such as *Lactobacillus*, *Bifidobacterium*, *Streptococcus*, *Pediococcus*, and *Ascophyllum*, also induced changes in expressions of GABA_A_ and GABA_B_ receptors^22,25^ in both heathy and disease mouse models of epilepsy, Parkinson’s disease, and autism with improvement of those disease conditions.

Although it is well accepted that prebiotics and probiotics have the ability in enhancing GABA production, based on our literature review, evidence for the direct GABA-increasing action of prebiotics is scarce relative to probiotics mentioned above. A study demonstrated that dietary FOS and inulin slightly, but not significantly, increased cecal GABA levels in mice, whereas their combinations with a *Lactiplantibacillus* probiotic significantly elevated cecal GABA levels higher than the prebiotic or the probiotic alone^26^. In our previous study, we demonstrated that dietary *Aspergillus*-derive protease, a substrate having prebiotic-like effects, markedly increased cecal GABA levels with an increased abundance of GABA-producing *Lactobacillus* and *Bifidobacterium*^27^. A combination of FOS and xylooligosaccharides prebiotics increased and restored GABA levels in cortex of acrylamide-induced oxidative-impaired rats^28^. On the other hand, a study demonstrated that a supplementation of GOS or a mix of FOS and GOS decreased GABA levels in cortex of APP/PS1 Alzheimer’s disease mice^29^. Some studies demonstrated that FOS or a mix of FOS and GOS altered expression of GABA_B_ receptors in hippocampus of pigs and mice^42,43^. Our present findings added a piece of evidence indicating the ability of prebiotics in increasing both gut and brain GABA levels with a positive association between gut GABA and brain GABA (Figs. 1 and 7).

Regarding changes in gut microbiota due to the prebiotic FOS, AL, or AP treatment in our present study, *Parabateroides*, *Akkermansia, Muribaculum, Hungatella*, *Marvinbryantia, Flavonifractor*, and *Incertae_sedis* genera are positively correlated with peripheral and brain GABA and homocarnosine levels, while *Blautia*, *Unclassified_Lachnospiraceae*, *Colidextribacter*, *Acetatifactor*, *Roseburia*, *Unclassified_Oscillospiraceae*, *Romboutsia*, and *Eubacterium_coprostanoligenes* genera are negatively correlated with peripheral and brain GABA and homocarnosine levels. *Clostridium_sensu_stricto_1* and *Lachnospiraceae_A2* genera exhibited both positive and negative correlations with peripheral and brain GABA and homocarnosine levels. Studies suggests that harboring glutamate decarboxylase (*gadB*), an enzyme converting glutamate to GABA, is the important characteristic of GABA-producing bacteria^15,44^. Integrated Microbial Genomes/Human Microbiome Project database demonstrated that *Bacteroides* (31.7%) was the most prevalent genus processing *gadB*, followed by *Escherichia* (22.5%), *Fusobacterium* (9.9%), *Providencia* (7.0%), *Parabacteroides* (4.9%), *Clostridium* (2.8%), *Bifidobacterium* (2.1%), *Lactobacillus* (2.1%), *Eggerthella* (2.1%), *Alistipes* (2.1%), *Listeria* (1.4%), *Oxalobacter* (1.4%), and others (9.9%)^15^. The *Akkermansia* genus was also shown to harbor *gadB* and be able to produce GABA strongly^45^. An increased abundance of *Akkermansia* was found to have a positive correlation with increased gut GABA levels in essential tremor mice treated a *Lactobacillus* probiotic^17^. Together with *Parabacteroides* in ketogenic diet, *Akkermansia* was found to increase brain GABA levels with protection against seizure^46^. In addition to *Parabacteroides* and *Akkermansia*, *Muribaculum* was demonstrated to have a positive association with blood and brain GABA levels under probiotic treatments^19,21^. For genera *Hungatella*, *Marvinbryantia, Flavonifractor*, and *Incertae_sedis*, the information of their relationship with peripheral and brain GABA levels is very limited. Taken together, the aforementioned results support our finding that *Parabacteroides*, *Akkermansia*, and *Muribaculum* are possibly the GABA producers exhibiting a positive correlation with gut and brain GABA levels under the prebiotic FOS and enzyme treatments.

For genera *Blautia*, *Unclassified_Lachnospiraceae*, *Colidextribacter*, *Acetatifactor*, *Roseburia*, *Unclassified_Oscillospiraceae*, *Romboutsia*, *Eubacterium_coprostanoligenes*, *Clostridium_sensu_stricto_1*, and *Lachnospiraceae_A2*, their association with GABA production is varied. *Blautia* has been proposed to be able to produce GABA via putrescine aminotransferase, an enzyme converting putrescine to GABA, and *Blautia* spp. was found to have a positive correlation with luminal GABA concentrations in piglets^47^. On the other hand, an increased abundance of *Blautia* with a decrease in serum GABA levels were found in insomnia rats^48^. *Lachnospiraceae* was found dominant in healthy people who had low fecal GABA levels^49^ and exhibited a negative correlation with brain GABA levels in probiotic-treated mice^17^, whereas a higher abundance of *Lachnospiraceae* was found along with higher brain GABA levels and higher GABA/glutamate ratio in Parkinson’s disease and epilepsy mouse models under probiotic treatments^21,50^. *Colidextribacte*r abundance had negative correlations with human-fecal and mouse-brain GABA levels^51,52^, but had a positive relationship with rat brain GABA levels^53^. *Acetatifactor* abundance was found to have negative correlations with levels of mouse brain GABA and hamster fecal N-arachidonoyl GABA, a GABA derivative^54,55^. *Roseburia* abundance showed positive correlations with human fecal and mouse brain GABA levels and a negative relationship with blood GABA levels of SAMP8 mice^56,57^. The *Oscillibacter* genus of the *Oscillospiraceae* family exhibited a negative correlation with gut and brain GABA levels in *Lactobacillus*-probiotic treated mice^17^, while its increased abundance was found with increased blood GABA levels in diabetic mice treated a plant-derived compound^58^. *Romboutsia* and *Clostridium_sensu_stricto_1* were found positively correlated with hippocampal GABA levels in diabetic rats with cognitive dysfunction^59^. An increased abundance of *Romboutsia* and a decreased abundance of *Clostridium_sensu_stricto* were found with a decrease in serum GABA levels in rats with insomnia^48^. The abundance of *Clostridium_sensu_stricto_1* and serum GABA were found negatively associated with childhood overweight/obesity^60^. *Eubacterium* has been reported to be both a GABA producer and consumer^44^. *Eubacterium_coprostanoligenes* exhibited a positive correlation with serum GABA in diabetic mice^58^, while its decreased abundance was found with an increase in serum GABA levels after transplantation of healthy fecal microbiota to children with autism spectrum disorder, with the improvement of gastrointestinal symptoms and autism-like behaviors^61^. Taken together, *Blautia*, *Unclassified_Lachnospiraceae*, *Colidextribacter*, *Acetatifactor*, *Roseburia*, *Unclassified_Oscillospiraceae*, *Romboutsia*, *Eubacterium_coprostanoligenes*, *Clostridium_sensu_stricto_1*, and *Lachnospiraceae_A2* are possibly involved in both GABA production and consumption under the prebiotic FOS and enzyme treatments.

Homocarnosine (GABA-L-histidine) is a brain-specific GABA-containing peptide. In our present study, we found that prebiotics could increase not only gut and brain GABA levels but also the brain-specific GABA-containing peptide homocarnosine. Our study proposed that in addition to GABA, brain GABA-related compounds, such as homocarnosine, may be potential mediators in transmitting beneficial effects of gut microbiota in the brain. Studies demonstrated that low levels of homocarnosine and its constituent amino acid GABA were often found in various neurological disorders such as depression, epilepsy, and Alzheimer’s disease^3,4,6,7,62^. In line with this, our recent study demonstrated that pre-aging mice with homocarnosine deficiency exhibited hyperactivity-, anxiety-, and depression-like behaviors^63^. More details regarding the potentials of GABA and homocarnosine as the mediators in the gut–brain axis and their potentials in brain disease prevention can be found in a previous work^9^ and the references therein.

Further studies are necessary to determine how gut-microbiota-derived GABA induces elevation of brain GABA and homocarnosine, whether direct or indirect way. Despite the long belief of its inability to cross blood-brain barrier (BBB), GABA has recently showed controversial BBB permeability wherein result is dependent on the analytical method used^64^. Aside from BBB, GABA elevation can be induced in other ways—such as vagus nerve and hormonal pathways. For the vagus nerve pathway, an increase in both GABA and total GABA (including homocarnosine) in the cerebrospinal fluid as a result of electrical stimulation of the vagus nerve in the chest and neck of epileptic patients has been demonstrated^65,66^. Studies showed that gut GABA could cross the intestinal barrier via H+/GABA symporter^67^ and subsequently interact with GABA receptors and transporters that are widely expressed on enteric neurons and vagus afferents^68^. For hormonal pathway, exogenous testosterone hormone supplementation (applied intramuscularly or trans-dermally) in trans men was found to induce changes in brain GABA levels (GABA plus homocarnosine)^69^, while oral intake of GABA could regulate hormone secretion, such as increasing plasma growth hormone and prolactin levels^70,71^.

There are two limitations in this study. First, we only used adolescent mice (postnatal days P35-63) for 4-weeks supplementations in this study. Second, this study tested the effects of FOS and the enzymes only in outbred ICR mice. In the future, it is of interest to determine if FOS and the enzymes can modulate composition of gut microbiota and increase gut and brain GABA levels in mice at different ages, such as adult and aging, and in other mouse strains.

In conclusion, our findings show that prebiotics and food factors with prebiotic-like effects mediate in the gut and brain connection by inducing elevation of brain GABA and homocarnosine levels as a result of alteration in gut microbiota composition. To the best of our knowledge, this study is a novel demonstration on the ability of dietary factors, other than probiotics, to induce brain GABA and homocarnosine production.

## Medthods

### Animals

A total of 32 male adolescent ICR mice (4 weeks old) were purchased from Charles River Japan, Hino, Japan. After a prior 7-day acclimatization with a non-purified commercial rodent diet (MF, Oriental Yeast, Tokyo, Japan), those mice (5 weeks old) were randomly divided into four groups (*n* = 8/group) to receive FOS supplementation or each enzyme supplementation mentioned below. All mice were maintained in accordance with the Guide for the Care and Use of Laboratory Animals established by Hiroshima University and approved by the Ethics Committee of the University (Ethical Approval No. C22-31-2). All mice were housed in a temperature-controlled room (24 ± 1 °C) in a 12-h light/dark cycle (lights on from 08:00–20:00 h) with free access to food and drinking water.

### FOS and enzyme supplementations

After the 7-day acclimatization, each experimental group was given with different diets for 4 weeks: the high-fat diet (HFD) Ctrl group; the AP group (HFD + 0.2%*(w/w) Aspergillus*-derived protease enzyme (Protease A “Amano” SD, Amano Enzyme Inc. Nagoya, Japan)); the AL group (HFD + 0.2%*(w/w) Aspergillus*-derived lipase enzyme (Lipase AP12, Amano Enzyme Inc)); and the FOS group (HFD + 10%*(w/w)* FOS (Fujifilm Wako Pure Chemical Corporation, Osaka, Japan)) (Fig. S1 and Table S1). The diets were changed freshly every 2 days, and body weight and food intake were measured every 2 days.

### Blood and tissue collections

At the end of the experiment, all mice were fasted for 6 h before being sacrificed under isoflurane anesthesia (between 13:00 and 16:00). Blood from abdominal veins was collected into tubes containing the anticoagulant heparin. Plasma was then obtained by centrifugation at 3000 × *g* for 10 min and stored at −80 °C until analysis. Cecal contents, fat tissues, and tibialis anterior skeletal muscle tissues were harvested immediately, weighed, snapped frozen in liquid nitrogen, and stored at −80 °C until analysis. Then, brain tissues of cortex, hippocampus, and hypothalamus were collected according to previous reports^72–74^ and shown in Fig. S2.

### GABA and homocarnosine determination by UPLC-MS/MS

Brain tissues and cecum content were homogenized in methanol with an internal standard (20 μM methionine sulfone (MS)) as previously reported^75,76^. Then, supernatants were evaporated to dryness and resuspended in methanol prior to analysis in UPLC-MS/MS (Waters, Milford, MA). For plasma samples, plasma was mixed with 20 μM-MS containing methanol at a ratio of 1:3 of plasma to methanol, followed by centrifugation, filtration, and injection to UPLC-MS/MS. Liquid chromatography was performed at 30 °C using an Acquity UPLC BEH C18 (1.7 μm, 2.1 × 50 mm) column (Waters) and a gradient system consisting of mobile phase A (5 mM perfluoroheptanoic acid (PFHpA; Sigma-Aldrich, Louis, MO)) in Milli-Q water and mobile phase B (5 mM PFHpA in methanol) at a flow rate of 400 μL/min. The gradient program was set at the following conditions: 0–0.5 min, 5–40% B; 0.5–10.5 min, 40–50% B; 10.5–11 min, 50–100% B; 11–12 min, 100% B; 12–12.5 min, 100–5% B; and 12.5–17.5 min, 5% B. Total run-to-run time was 17.5 min with an injection volume of 5 μL. Mass spectrometric analysis was performed by multiple reaction monitoring (MRM) in the ESI-positive mode, 400 °C desolvation temperature, 120 °C source temperature, nitrogen as desolvation and cone gas, and argon as the collision gas for MRM and daughter-ion scans. The details of the capillary voltage, cone voltage, and MRM for each of compounds were established as reported previously^77^. Homocarnosine and GABA standards were purchased from Cosmo Bio International (Tokyo, Japan) and Nacalai Tesque (Kyoto, Japan), respectively.

### Microbiome analysis by 16S rRNA gene sequencing

Cecal content samples were used for analysis. Bacterial DNA extraction, 16S ribosomal RNA gene sequencing, and all bioinformatic analyses were performed by Shanghai Biozeron Biotechnology Co., Ltd. (Shanghai, China). DNA was extracted using a fecal genomic DNA extraction kit from Solarbio (Beijing, China) and DNA concentrations were measured using Synergy H1 (Bio-Tek) following the manufacturer’s standard protocols. The first PCR was performed using the primers 341F (5′-CCTAYGGGRBGCASCAG-3′) and 805 R (5′-GGACTACNNGGGTATCTAAT-3′). Barcoded V3–V4 PCR amplicons were sequenced using an Illumina MiSeq platform at 2 × 250 bp. Sequencing data were processed and analyzed using Quantitative Insights into Microbial Ecology (QIIME2 (v. 2023.2)). Following demultiplexing, the sequences underwent quality filtering (quality scores over than 25), and chimeric sequences were removed. The statistical summary of the high-throughput amplicon sequencing data is detailed in Table S2. The remaining reads were then merged (average sequence length: 418 bp) using the DADA2 plugin to generate amplicon sequence variants (ASVs). Taxonomic classification of the ASVs was performed using the q2-feature-classifier plugin, with reference to the SILVA 138 database at 99% similarity. For diversity analysis, the sequences were rarefied to 12,451 reads per sample to ensure uniform sequencing depth across samples.

### Statistical analysis

All values are expressed as the means ± SEM. A two-tailed unpaired *t*-test or an one-way ANOVA followed by Dunnett’s multiple comparisons was used to test significances between groups. Correlation analysis was computed using a nonparametric Spearman correlation (two-tailed) under correlation matrix in GraphPad Prism 10 to determine the correlative relationship. All statistical analyses were done by using GraphPad Prism 10 (GraphPad Software, CA, USA). *P* < 0.05 indicated statistical significance. For microbiome analysis, data separation in the principal coordinate analysis (PCoA) ordination of beta diversity was tested using the PERMANOVA permutation-based statistical test in vegan-R, and *p*-values were generated based on 999 permutations using the “beta-group-significance” plugin in QIIME2. The bacterial taxa data were subjected to linear discriminant analysis effect size (LEfSe), which uses the two-tailed nonparametric Kruskal–Wallis test to evaluate the significance of differences between taxa. The number of animals in each experiment is stated in the respective figure captions.

## Supplementary information

Supplementary Information - PDF

## Data Availability

All other data are available from the authors upon request.
