# Supplementary material for: Fructooligosaccharides and Aspergillus enzymes increase brain GABA and homocarnosine by modulating microbiota in adolescent mice
Source: NPJ Sci Food. 2025 Apr 3;9:48. doi: 10.1038/s41538-025-00383-1 (PMC11965294; doi:10.1038/s41538-025-00383-1)
Supplement: Supplementary file 1 — Supplementary Information - PDF [file 41538_2025_383_MOESM1_ESM.pdf]

Supplementary Figure and Table

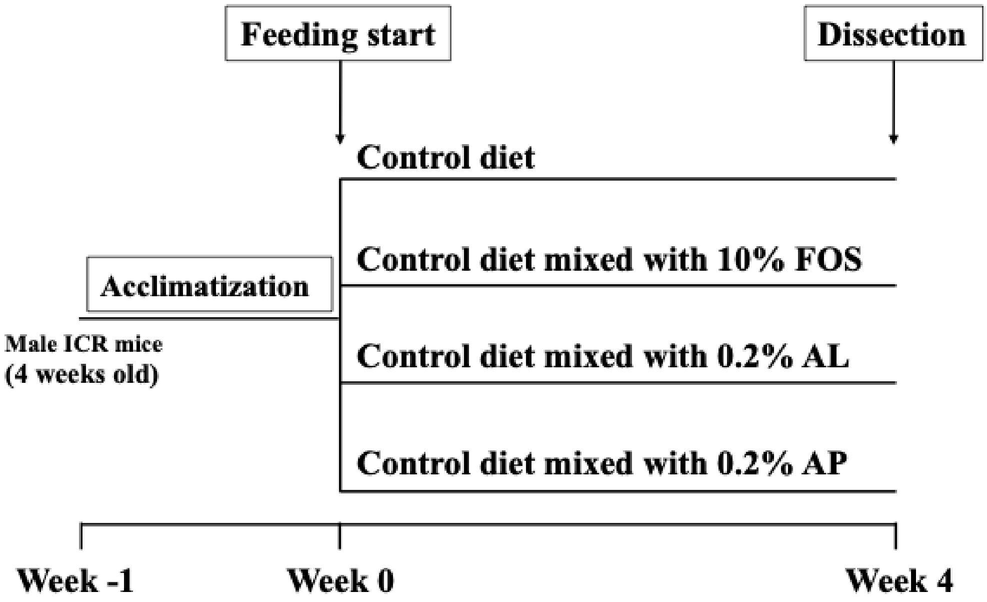

Figure S1. The experimental scheme.
FOS, fructo-oligosaccharides; AL, *Aspergillus*-derived lipase enzyme (Lipase AP12, Amano Enzyme Inc); AP, *Aspergillus*-derived protease enzyme (Protease A "Amano" SD, Amano Enzyme Inc. Nagoya, Japan)

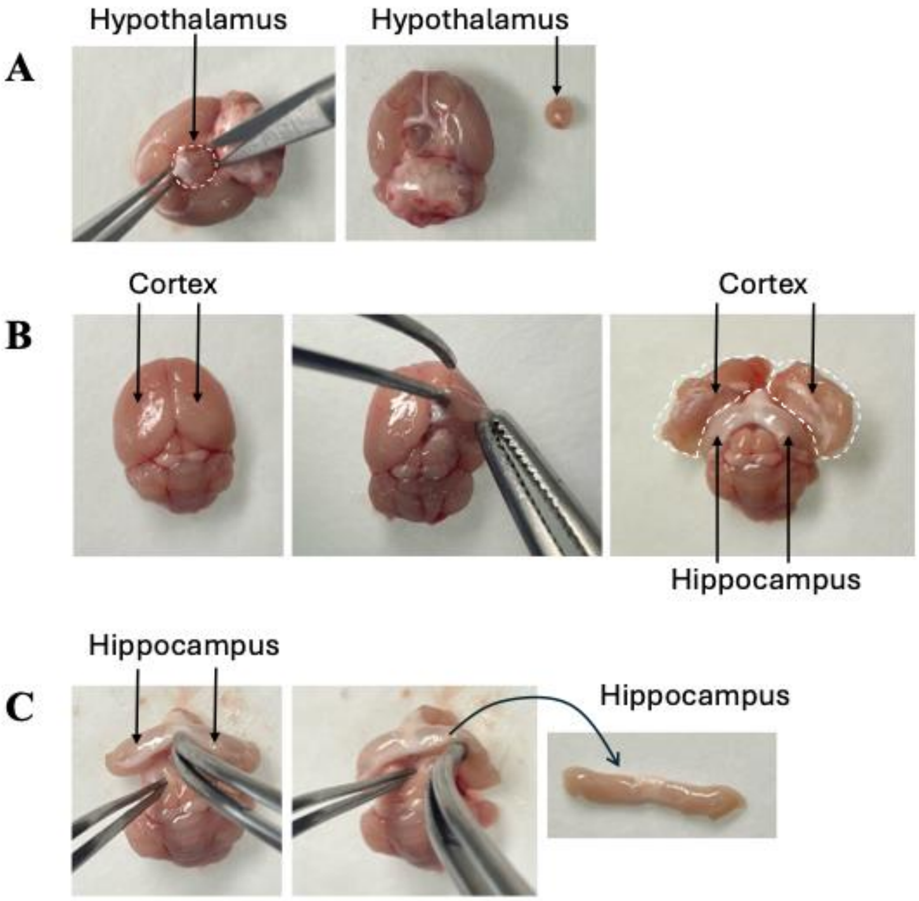

Figure S2. Dissection of the hypothalamus, cortex, and hippocampus from the mouse brain
The brain was dissected on an ice-cold plate. Brain tissues of hypothalamus (A), cortex (B), and hippocampus (C) were dissected and collected using a tissue forceps and a micro scissor. The brain regions were collected according to previous reports shown below.

- Spijker S. Dissection of rodent brain regions. *Neuroproteomics*. 2011:13-26.
- Basil P, Li Q, McAlonan GM, Sham PC. Genome-wide DNA methylation data from adult brain following prenatal immune activation and dietary intervention. *Data in brief*. 2019;26:104561.
- Laukoter S, Amberg N, Pauler FM, Hippenmeyer S. Generation and isolation of single cells from mouse brain with mosaic analysis with double markers-induced uniparental chromosome disomy. *STAR protocols*. 2020;1(3):100215.

Table S1. Experimental diet composition

Diet composition	Control	Prebiotics		
		FOS	AL	AP
Beef tallow	30%	30%	30%	30%
Casein	25%	25%	25%	25%
Vitamin mixture[1]	1%	1%	1%	1%
Mineral mixture[1]	3.5%	3.5%	3.5%	3.5%
Cellulose	5%	5%	5%	5%
Sucrose	20%	20%	20%	20%
Corn starch	15.5%	5.5%	14.83%	15.3%
AP[2]	-	-	-	0.2%
AL[3]	-	-	0.67%	-
FOS[4]	-	10%	-	-

[1]American Institute for Nutrition (AIN-93)
[2]AP: *Aspergillus*-derived protease enzyme (Protease A "Amano" SD)
[3]AL: *Aspergillus*-derived lipase enzyme (Lipase AP12), the enzyme powder contains 70% dextrin
[4]FOS: Fructooligosaccharides

Table S2. The high-throughput sequence statistics were generated using QIIME2

Sample	Raw Sequence Reads	Filtered Sequences	Denoised Sequences	Merged Sequences	Non-chimeric Sequences
Ctrl1	41165	39854	39617	38219	36464
Ctrl2	37444	36447	36236	35160	32289
Ctrl3	35018	34233	34049	32655	21979
Ctrl4	38246	36931	36678	34530	19037
Ctrl5	40242	39352	39256	38389	35921
Ctrl6	33633	32839	32688	31820	31704
Ctrl7	40341	39349	39061	36726	12451
FOS1	36661	35840	35663	34940	32055
FOS2	37768	36761	36572	35954	27751
FOS3	36241	35419	35166	33655	25781
FOS4	35163	34257	34042	31543	17417
FOS5	37662	36787	36642	35230	29652
FOS6	36469	35571	35413	34949	26993
FOS7	41842	40915	40660	38684	13165
AL1	36116	35299	35032	33483	21206
AL2	38876	37997	37779	36405	25392
AL3	38957	38082	37817	34007	22845
AL4	35509	34706	34447	32589	28698
AL5	36206	35409	35136	33772	32223
AL6	40889	40049	39765	37464	33553
AL7	33929	33189	32876	30886	29571
AP1	34359	33616	33416	31881	23821
AP2	40193	39295	39032	35939	19833
AP3	36098	35314	34807	31084	13433
AP4	40321	39433	39294	38499	35824
AP5	38321	37332	37040	32783	20672
AP6	41407	40407	40120	36103	26046
AP7	37536	36591	36369	33763	24614
AP8	40423	39401	39161	36606	33509
**Total**	**1097035**	**1070675**	**1063834**	**1007718**	**753899**

Ctrl: Control, FOS: fructo-oligosaccharides, AL: *Aspergillus*-derived lipase, and AP: *Aspergillus*-derived protease.
